# Time-at-bedside and competency acquisition: a secondary analysis of GM-ITE domain scores in Japanese resident physicians

**DOI:** 10.1186/s12909-025-08076-9

**Published:** 2025-11-03

**Authors:** Kohta Katayama, Toshihiko Takada, Yuji Nishizaki, Kazuya Nagasaki, Taro Shimizu, Yu Yamamoto, Takashi Watari, Yasuharu Tokuda, Vineet Chopra, Yoshiyuki Ohira

**Affiliations:** 1 Department of General Internal Medicine, St. Marianna University School of Medicine, Kanagawa, Japan; 2 Department of Clinical Epidemiology, Graduate School of Medicine, Fukushima Medical University, Fukushima, Japan; 3 Department of General Medicine, Shirakawa Satellite for Teaching and Research (STAR), Fukushima Medical University, Fukushima, Japan; 4 Division of Medical Education, School of Medicine, Juntendo University, Tokyo, Japan; 5 Department of Internal Medicine, Mito Kyodo General Hospital, University of Tsukuba, Ibaraki, Japan; 6 Department of Diagnostic and Generalist Medicine, Dokkyo Medical University Hospital, Tochigi, Japan; 7 Division of General Medicine, Center for Community Medicine, Jichi Medical University, Tochigi, Japan; 8 Integrated Clinical Education Center, Kyoto University Hospital, Kyoto, Japan; 9 Muribushi Okinawa Center for Teaching Hospitals, Urasoe, Okinawa Japan; 10 Department of Medicine, University of Colorado School of Medicine, Aurora, USA

**Keywords:** Competency, Time-at-bedside, Clinical residency, Japan

## Abstract

**Background:**

Direct bedside learning is recognized as essential for clinical skill development, yet its domain-specific effects on competency acquisition have not been fully elucidated. We examined how self‐reported time‐at‐bedside was associated with performance across four GM‐ITE competency domains.

**Methods:**

We performed a nationwide multicenter, cross-sectional study of Japanese first- and second-year postgraduate resident physicians who took the General Medicine In-Training Examination (GM-ITE) in late 2022. Time-at-bedside was defined as the average self-reported time per day a resident spent providing direct care at the patients’ bedside and was stratified into six categories: C1 (10–20 min/day), C2 (30–50 min/day), C3 (60–80 min/day), C4 (90–110 min/day), C5 (120–140 min/day), and C6 (≥ 150 min/day). Data on time-at-bedside were collected through an electronic survey conducted immediately after the GM-ITE. A linear mixed-effects model was employed to examine the association between time-at-bedside and four GM-ITE competency-specific scores—medical interview and professionalism (MP), symptomatology and clinical reasoning (CR), physical examination and clinical procedures (PP), and disease knowledge (DK).

**Results:**

Of 5,344 residents analyzed, more time-at-bedside showed only weak associations with MP and CR scores. In contrast, PP and DK scores increased in a dose–response pattern. Compared to C1, PP adjusted score differences were 0.3 (95% confidence interval [95% CI]: 0.07 to 0.48) in C2, 0.5 (95% CI: 0.27 to 0.73) in C3, 0.6 (95% CI: 0.13 to 1.01) in C5. Similarly, DK adjusted score differences were 0.6 (95% CI: 0.23 to 0.94) in C2, 0.5 (95% CI: 0.18 to 0.96) in C3, and 0.6 (95% CI: 0.16 to 1.66) in C5.

**Conclusion:**

In Japanese clinical residency, more time-at-bedside was associated with the acquisition of physical examination skills, clinical procedure skills, and disease knowledge. Future prospective longitudinal cohort studies are warranted to determine whether more time-at-bedside can accelerate these competencies.

**Supplementary Information:**

The online version contains supplementary material available at 10.1186/s12909-025-08076-9.

## Introduction

Cultivating clinical proficiency depends not only on classroom instruction but on routine, hands-on bedside encounters during residency [1–7]. By engaging directly with patients, residents actively hone their communication, develop nuanced diagnostic hypotheses, and strengthen their clinical reasoning processes. Frequent bedside practice embeds procedural techniques into memory and places pathophysiological concepts in real-world context. Indeed, one previous study reported correct diagnoses in over two-thirds of cases when physicians performed full bedside assessments [8]. Yet mounting evidence indicates that trainees now spend fewer hours at the bedside—an erosion blamed on electronic records, work-hour limits, and streamlined hospital stays—which may undermine the formation of critical clinical skills [2–7].

Since its inception in 2012, the General Medicine In-Training Examination (GM-ITE) has functioned as a standardized assessment of Japanese resident physicians’ proficiency across four core competency domains—medical interview and professionalism, clinical reasoning and symptomatology, physical examination and clinical procedures, and foundational disease knowledge—that align with Japan’s postgraduate medical education (PGME) competency framework [9]. By providing quantifiable domain-specific scores, the GM-ITE now underpins comparative evaluations of educational environments and residency program effectiveness throughout the country [10, 11].

Building on our previous findings of a positive association between self-reported time-at-bedside and overall GM-ITE performance [12], the specific influence of time-at-bedside on each competency domain remains unclear. Understanding these domain-specific associations is essential for designing targeted educational strategies to enhance specific aspects of clinical training. This study aims to investigate the association between self-reported time-at-bedside and the four GM-ITE competencies. By analyzing the specific influences on each competency domain, we seek to provide evidence-based recommendations for optimizing residency programs and improving educational outcomes.

## Methods

### Study design and population

Building on our nationwide GM-ITE dataset, we conducted a secondary analysis to explore how self-reported time-at-bedside relate to competency domain-specific GM-ITE performance among Japanese resident physicians [12]. Ethical approval was secured from the Japan Institute for Advancement of Medical Education Program Ethics Review Board (JAMEP 22 − 9), and all participants provided written informed consent under the Japanese Ethical Guidelines for Medical and Health Research involving Human Subjects and the Helsinki Declaration.

Eligibility was limited to PGY-1 and PGY-2 resident physicians, who completed both the GM-ITE and the accompanying electronic clinical training environment survey at the end of the 2022 academic year. We omitted resident physicians who either declined participation or left incomplete responses in the survey.

### Exposure: self-reported time-at-bedside

Participants reported their average daily time-at-bedside via an electronic clinical training environment survey administered immediately after the GM-ITE. Specifically, residents indicated the number of minutes per day spent at patients’ bedsides during routine duty hours, choosing from sixteen 10-minute increments (10 to ≥ 150 min). Self-reported time-at-bedside was collapsed into six 30-minute interval groups, consistent with the previous study [12]: C1 (10–20 min/day), C2 (30–50 min/day), C3 (60–80 min/day), C4 (90–110 min/day), C5 (120–140 min/day), C6 (≥ 150 min/day). This categorization reflects the typical Japanese resident physicians’ number of inpatient in charge. They are in charge of five to ten inpatients, implying approximately 25–50 min of time-at-bedside if they spend five minutes at each patient bedside.

### Covariates and data sources

Our multivariable models included both hospital- and resident-level variables. Hospital-level variables included hospital type (community, university, or university branch) and annual ambulance transport volume (0–999; 1,000–1,999; 2,000–2,999; ≥3,000), obtained from the Medical Care Information Bureau (http://caremap.jp/). Resident-level variables comprised postgraduate year (PGY-1 or PGY-2), cumulative months of internal medicine training (0–5; 6–10; 11–15; 16–20; ≥21), general medicine department training, average number of inpatients in charge (0–4; 5–9; 10–14; ≥15; or unknown), weekly duty hours (59≤; 60–79; ≥ 80 h), daily self-study time (0–30; 31–60; 61–90; ≥91 min), and burnout status [12–14]. Burnout status was defined by a validated single-item measure: residents selecting any of the top three response categories (“totally burned out” to “beginning to burn out”) were classified as burned out (sensitivity 53.8%; specificity 88.2%) [15]. Covariate data—excluding ambulance transport volumes—were collected through an electronic clinical training environment survey [12].

### Outcome: GM-ITE competency scores

The primary outcomes of this analysis were the four competency domain-specific scores on the GM-ITE, a secure, computer-based examination consisting of 80 one-point multiple-choice items. Items are distributed as follows: medical interview and professionalism (MP; 8 questions), symptomatology and clinical reasoning (CR; 18 questions), physical examination and clinical procedures (PP; 18 questions), and disease knowledge (DK; 36 questions). Each question was developed by a committee of board-certified specialists and supervising physicians and underwent independent peer review to confirm content validity and assign it to the appropriate competency domain. The GM-ITE score has been reported to be associated with the Professional and Linguistic Assessments Board examination score, which assesses whether international medical graduates possess the essential skills and knowledge required to practice medicine in the United Kingdom [11]. In the 2022 administration, eight items employed video vignettes to enhance clinical realism; examples of these video-based questions appear in the Supporting Information (Supplementary Information: Video-Based Question Sample). Domain scores range from 0 to the total number of items in each category, with higher scores reflecting greater proficiency. These four competencies correspond to the PGME competency framework established by the Japanese Ministry of Health, Labour and Welfare [9].

### Statistical analyses

Descriptive statistics summarized hospital- and resident-level variables across six time-at-bedside categories using frequencies and percentages. We then fitted random-intercept and random-slope linear mixed models to estimate the adjusted score differences in each competency-specific GM-ITE score (MP, CR, PP, and DK) associated with time-at-bedside categories, while accounting for clustering at the hospital level [16]. All analyses were conducted as a complete-case analysis, excluding any records with missing values. Details of variable classification are provided in the Covariates and Data Sources section. Covariates included hospital- (hospital type and number of ambulance transports per year) and resident-level variables (postgraduate year, months of internal medicine training, general medicine department training, average number of inpatients in charge, weekly duty hours, daily self-study time, and burnout status). Adjusted score differences were calculated relative to the C1 category (10–20 min/day). We regarded two-tailed p-values below 0.05 as statistically significant. All analyses were performed in Stata/SE software, version 15 (Stata Corp. College Station, TX, USA).

## Results

Of the 8,438 PGY-1 and PGY-2 resident physicians who completed the 2022 GM-ITE, 5,344 from 606 hospitals were included in the final analysis after excluding those who declined participation (*n* = 2,857) or submitted incomplete survey data (*n* = 237). The distribution across self-reported time-at-bedside categories was C1: 25.0% (*n* = 1,335); C2: 41.6% (*n* = 2,222); C3: 24.6% (*n* = 1,315); C4: 4.3% (*n* = 230); C5: 3.0% (*n* = 159); and C6: 1.6% (*n* = 83) (Table 1).

**Table 1:** Summary of hospital and resident characteristics according to self-reported time-at-bedside categories

		Self-reported time-at-bedside*					
	Total	C1	C2	C3	C4	C5	C6
	*N* = 5,344	*N* = 1,335 (25.0)	*N* = 2,222 (41.6)	*N* = 1,315 (24.6)	*N* = 230 (4.3)	*N* = 159 (3.0)	*N* = 83 (1.6)
Hospital-level variables							
Hospital type							
Community	4,464 (82.5)	1,141 (25.6)	1,847 (41.4)	1,102 (24.7)	184 (4.1)	124 (2.8)	66 (1.5)
University	533 (10.0)	130 (23.5)	232 (43.5)	126 (23.6)	18 (3.4)	19 (3.6)	8 (1.5)
University branch	347 (6.5)	64 (18.4)	143 (41.2)	87 (25.1)	28 (8.1)	16 (4.6)	9 (2.6)
Annual number of ambulance transports							
0 to 999	1,008 (18.9)	248 (24.6)	437 (43.4)	247 (24.5)	38 (3.8)	25 (2.5)	13 (1.3)
1,000 to 1,999	2,116 (39.6)	538 (25.4)	888 (42.0)	515 (24.3)	86 (4.1)	62 (2.9)	27 (1.3)
2,000 to 2,999	1,691 (31.6)	410 (24.2)	676 (40.0)	434 (25.7)	84 (5.0)	57 (3.4)	30 (1.8)
3,000 or more	529 (9.9)	139 (26.3)	221 (41.8)	119 (22.5)	22 (4.2)	15 (2.8)	13 (2.5)
Resident-level variables							
Postgraduate year							
PGY-1	2,760 (51.6)	692 (25.1)	1,145 (41.5)	681 (24.7)	124 (4.5)	85 (3.1)	33 (1.2)
PGY-2	2,584 (48.4)	643 (24.9)	1,077 (41.7)	634 (24.5)	106 (4.1)	74 (2.9)	50 (1.9)
IM department training							
0–5 months	1,431 (26.8)	350 (24.5)	609 (42.6)	336 (23.5)	65 (4.5)	49 (3.4)	22 (1.5)
6–10 months	3,294 (61.6)	855 (26.0)	1,359 (41.3)	804 (24.4)	139 (4.2)	87 (2.6)	50 (1.5)
11–15 months	540 (10.1)	111 (20.6)	227 (42.0)	150 (27.8)	23 (4.3)	21 (3.9)	8 (1.5)
16–20 months	57 (1.1)	13 (22.8)	18 (31.6)	20 (35.1)	2 (3.5)	2 (3.5)	2 (3.5)
21 or more months	22 (0.4)	6 (27.3)	9 (40.9)	5 (22.7)	1 (4.5)	-	1 (4.5)
GM department training	2,454 (45.9)	580 (23.6)	994 (40.5)	636 (25.9)	116 (4.7)	82 (3.3)	46 (1.9)
Average number of inpatients in charge							
0–4	2,068 (38.7)	733 (35.4)	871 (42.1)	369 (50.3)	42 (3.0)	33 (1.6)	20 (1.0)
5–9	2,663 (49.8)	519 (19.5)	1,132 (42.5)	736 (27.6)	154 (5.8)	88 (3.3)	34 (1.3)
10–14	387 (7.2)	37 (9.6)	149 (38.5)	140 (36.2)	28 (7.2)	21 (5.4)	12 (3.1)
15 or more	117 (2.2)	15 (12.8)	27 (23.1)	44 (37.6)	5 (4.3)	12 (10.3)	12 (10.3)
Unknown	109 (2.0)	31 (28.4)	43 (39.4)	26 (23.9)	1 (1.0)	5 (4.6)	3 (2.8)
Weekly duty hours							
59 or less	2,715 (50.8)	786 (30.0)	1,183 (43.6)	578 (21.3)	89 (3.3)	51 (1.9)	28 (1.0)
60 to 79	1,903 (35.6)	412 (21.7)	792 (41.6)	515 (27.1)	84 (4.4)	71 (3.7)	29 (1.5)
80 or more	726 (13.6)	137 (18.9)	247 (34.0)	222 (30.6)	57 (7.9)	37 (5.1)	26 (3.6)
Daily self-study time							
0–30 min	92 (1.7)	39 (42.4)	33 (35.9)	14 (15.2)	4 (4.3)	-	2 (2.2)
31–60 min	2,429 (45.5)	744 (30.6)	1,022 (42.1)	514 (21.2)	87 (3.6)	45 (1.9)	37 (1.5)
61–90 min	2,052 (38.4)	426 (20.8)	882 (43.0)	548 (26.7)	90 (4.4)	73 (3.6)	33 (1.6)
91 or more minutes	771 (14.4)	126 (16.3)	305 (39.6)	239 (31.0)	49 (6.4)	41 (5.3)	11 (1.4)
Burnout status	994 (17.7)	253 (25.5)	374 (37.6)	224 (22.5)	41 (4.1)	35 (3.5)	17 (1.7)

The numbers in parentheses in columns C1–C6 indicate the percentage of participants in each category for each variable

*IM* Internal medicine, *GM* General medicine, *PGY* Postgraduate year

*Self-reported time-at-bedside categories were as follows: ategory 1 (C1, 10–20 min per day), Category 2 (C2, 30–50 min per day), Category 3 (C3, 60–80 min per day), Category 4 (C4, 90–110 min per day), Category 5 (C5, 120–140 min per day), and Category 6 (C6, 150 min or more minutes per day). The numbers in parentheses in the “Total” column represent the percentage distribution of each hospital- and resident-level variable

**Fig. 1:**
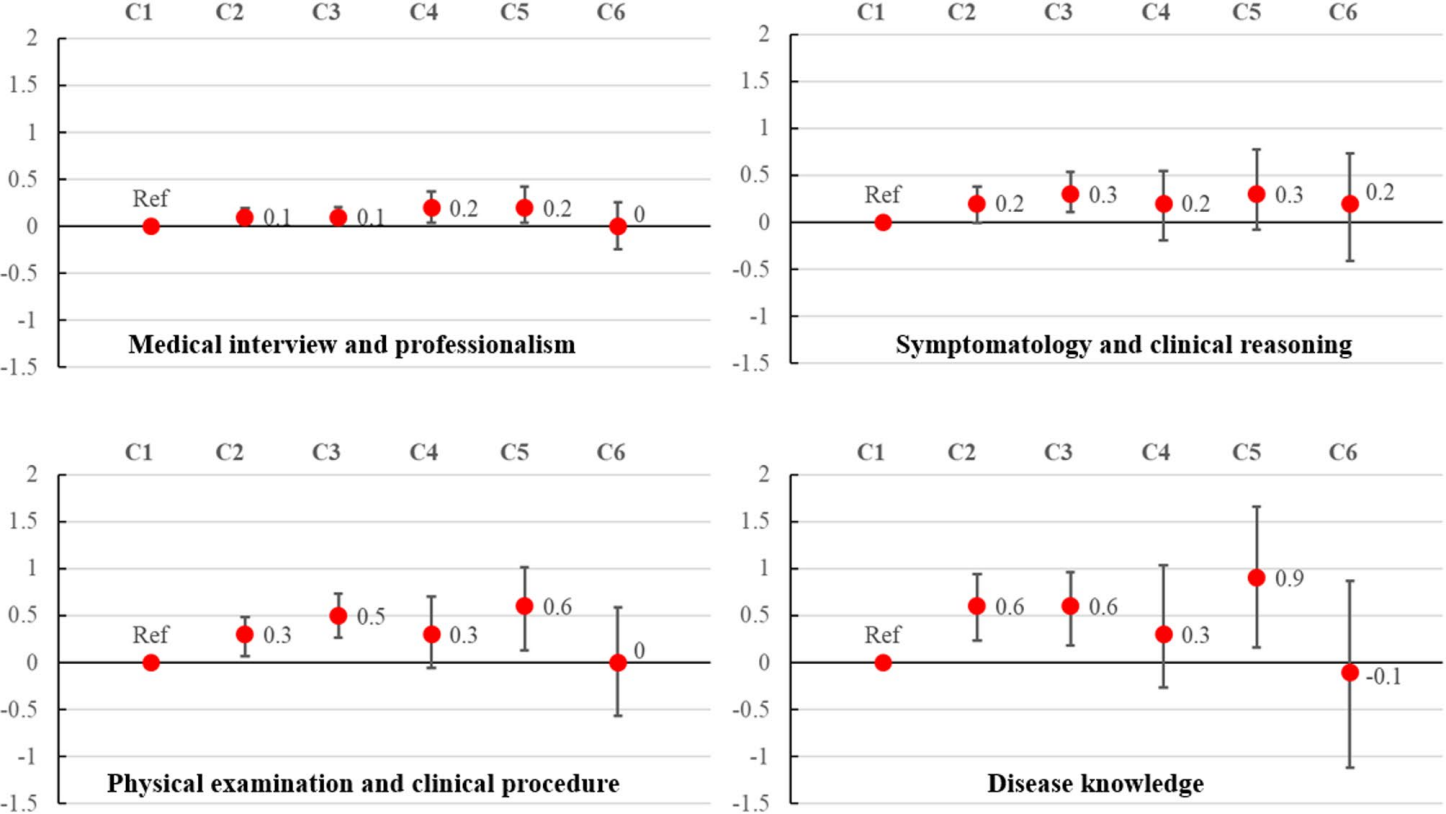
Adjusted score differences according to time-at-bedside categories and four competencies. The time-at-bedside categories were as follows: C1, 10–20 min/day; C2, 30–50 min/day; C3, 60–80 min/day; C4, 90–110 min/day; C5, 120–140 min/day; and C6, ≥ 150 min/day

In linear mixed-effects models adjusting for hospital- and resident-level variables (Fig. 1; Table 2), more self-reported time-at-bedside showed only modest positive associations with the MP domain. MP scores increased modestly across C2–C5 (adjusted score differences [aSD] = 0.1–0.2; 95% confidence intervals [CIs]: 0.02 to 0.42). In contrast, CR score increased significantly only in C3 (aSD = 0.3; 95% CI: 0.11 to 0.54).

**Table 2:** Association between self-reported time-at-bedside and each competency

Variables	MP		CR		PP		DK	
	aSD (95% CI)	*p*	aSD (95% CI)	*p*	aSD (95% CI)	*p*	aSD (95% CI)	*p*
Time-at-bedside*								
C1	Reference		Reference		Reference		Reference	
C2	0.1 (0.03 to 0.19)	< 0.01	0.2 (−0.01 to 0.38)	0.06	0.3 (0.07 to 0.48)	< 0.01	0.6 (0.23 to 0.94)	< 0.01
C3	0.1 (0.02 to 0.20)	0.02	0.3 (0.11 to 0.54)	< 0.01	0.5 (0.27 to 0.73)	< 0.01	0.6 (0.18 to 0.96)	< 0.01
C4	0.2 (0.04 to 0.37)	0.01	0.2 (−0.19 to 0.55)	0.34	0.3 (−0.06 to 0.70)	0.10	0.4 (−0.27 to 1.03)	0.26
C5	0.2 (0.04 to 0.42)	0.02	0.3 (−0.08 to 0.78)	0.11	0.6 (0.13 to 1.01)	0.01	0.9 (0.16 to 1.66)	0.02
C6	0 (−0.25 to 0.26)	0.94	0.2 (−0.41 to 0.73)	0.57	0 (−0.57 to 0.59)	0.97	0.1(−1.12 to 0.87)	0.80
Hospital-level variables								
Hospital type								
Community	Reference		Reference		Reference		Reference	
University	−0.2 (−0.27 to −0.06)	< 0.01	−0.7 (−0.96 to −0.40)	< 0.01	−1.0 (−1.28 to −0.68)	< 0.01	−1.7 (−2.23 to −1.20)	< 0.01
University branch	−0.3 (−0.48 to −0.22)	< 0.01	−1.1 (−1.40 to −0.74)	< 0.01	−1.3 (−1.60 to −0.91)	< 0.01	−2.0 (−2.62 to −1.43)	< 0.01
Annual number of ambulance transports								
0 to 999	Reference		Reference		Reference		Reference	
1,000 to 1,999	0 (−0.06 to 0.11)	0.56	0.2 (0.01 to 0.42)	0.04	0.3 (0.12 to 0.54)	< 0.01	0.4 (0.07 to 0.79)	0.02
2,000 to 2,999	0 (−0.06 to 0.12)	0.50	0.3 (0.07 to 0.51)	< 0.01	0.7 (0.48 to 0.93)	< 0.01	0.7 (0.30 to 1.08)	< 0.01
3,000 or more	0.1 (−0.05 to 0.20)	0.24	0.9 (0.59 to 1.20)	< 0.01	1.0 (0.72 to 1.36)	< 0.01	1.4 (0.82 to 1.92)	< 0.01
Resident-level variables								
Postgraduate year								
PGY-1	Reference		Reference		Reference		Reference	
PGY-2	0 (−0.09 to 0.05)	0.57	−0.2 (−0.33 to −0.02)	0.03	0.5 (0.37 to 0.68)	< 0.01	0.4 (0.13 to 0.66)	< 0.01
IM department training								
0–5 months	Reference		Reference		Reference		Reference	
6–10 months	0 (−0.05 to 0.10)	0.47	0.1 (−0.07 to 0.26)	0.26	0.2 (0.01 to 0.35)	0.04	0.4 (0.10 to 0.67)	< 0.01
11–15 months	0 (−0.08 to 0.17)	0.45	0.3 (0.05 to 0.59)	0.02	0.1 (−0.13 to 0.42)	0.29	0.6 (0.18 to 1.11)	< 0.01
16–20 months	−0.2 (−0.48 to 0.12)	0.24	0.3 (−0.33 to 0.99)	0.33	0.4 (−0.27 to 1.06)	0.25	1.1 (−0.06 to 2.21)	0.06
21 or more months	−0.1 (−0.57 to 0.38)	0.69	0.2 (−0.82 to 1.25)	0.69	0.2 (−0.87 to 1.21)	0.75	0.4 (−1.41 to 2.12)	0.69
GM department training								
No	Reference		Reference		Reference		Reference	
Yes	0.1 (0.03 to 0.16)	< 0.01	0.1 (−0.08 to 0.20)	0.40	0.2 (0.06 to 0.36)	< 0.01	0.3 (0.02 to 0.51)	0.03
Average number of inpatients in charge								
0–4	Reference		Reference		Reference		Reference	
5–9	0 (−0.08 to 0.06)	0.76	0.4 (0.26 to 0.56)	< 0.01	0.1 (−0.003 to 0.30)	0.05	0.8 (0.54 to 1.06)	< 0.01
10–14	0 (−0.12 to 0.13)	0.92	0.6 (0.35 to 0.92)	< 0.01	0.1 (−0.16 to 0.42)	0.38	0.9 (0.45 to 1.43)	< 0.01
15 or more	0.1 (−0.08 to 0.35)	0.23	0.4 (−0.12 to 0.84)	0.14	0.2 (−0.31 to 0.67)	0.47	0.9 (0.10 to 1.77)	0.03
Unknown	−0.1 (−0.30 to 0.13)	0.45	0 (−0.49 to 0.47)	0.97	−0.2 (−0.73 to 0.23)	0.32	0.4 (−0.41 to 1.22)	0.33
Weekly duty hours								
59 or less	Reference		Reference		Reference		Reference	
60–79	0 (−0.03 to 0.10)	0.33	0.2 (0.07 to 0.36)	< 0.01	0.2 (0.09 to 0.39)	< 0.01	0.6 (0.37 to 0.88)	< 0.01
80 or more	0 (−0.11 to 0.08)	0.76	0.1 (−0.08 to 0.34)	0.23	0.2 (−0.03 to 0.39)	0.10	0.3 (−0.04 to 0.68)	0.08
Self-study time per day								
0–30 min	Reference		Reference		Reference		Reference	
31–60 min	0.2 (−0.04 to 0.44)	0.10	0.04 (−0.48 to 0.55)	0.89	0.6 (0.10 to 1.13)	0.02	0.3 (−0.59 to 1.16)	0.53
61–90 min	0.2 (0.01 to 0.48)	0.04	0.3 (−0.18 to 0.86)	0.20	0.9 (0.33 to 1.37)	< 0.01	0.8 (0.07 to 1.70)	0.07
91 or more minutes	0.2 (−0.03 to 0.46)	0.09	0.5 (−0.08 to 0.99)	0.10	1.1 (0.53 to 1.61)	< 0.01	1.2 (0.24 to 2.07)	0.01
Burnout status								
No	Reference		Reference		Reference		Reference	
Yes	−0.1 (−0.17 to −0.01)	0.02	−0.1 (−0.26 to 0.09)	0.34	0 (−0.18 to 0.17)	0.94	−0.4 (−0.70 to −0.10)	< 0.01

Multi-level analysis was performed to estimate score differences of each competency after adjusting for hospital type, number of ambulance transports per year, postgraduate year, IM department training, GM department training, number of inpatients in charge, duty hours per week, self-study time per day, and burnout

*aSD* Adjusted score difference, *CI* Confidence interval, *CR* Symptomatology and clinical reasoning, *DK* Disease knowledge, *IM* Internal medicine, *GM* General medicine, *GM-ITE* General Medicine In-Training Examination, *MP* Medical interview and professionalism *PGY* Postgraduate year, *PP* Physical examination and clinical procedures

*The time-at-bedside categories were as follows: Category 1 (C1, 10–20 min per day), Category 2 (C2, 30–50 min per day), Category 3 (C3, 60–80 min per day), Category 4 (C4, 90–110 min per day), Category 5 (C5, 120–140 min per day), and Category 6 (C6, 150 min or more minutes per day)

In contrast, PP and DK scores demonstrated stepwise increases with more time-at-bedside (C2, C3, and C5) (Fig. 1; Table 2). PP score increased at C2 (aSD = 0.3; 95% CI: 0.07 to 0.48), C3 (aSD = 0.5; 95% CI: 0.27 to 0.73), and C5 (aSD = 0.6; 95% CI: 0.13 to 1.01). Similarly, DK score increased at C2 (aSD = 0.6; 95% CI: 0.23 to 0.94), C3 (aSD = 0.6; 95% CI: 0.18 to 0.96), and C5 (aSD = 0.9; 95% CI: 0.16 to 1.66). We used the aSDs between PGY-2 and PGY-1 residents (0.5 points for PP; 0.4 points for DK) as benchmarks for clinical meaningfulness and found that the aSDs at C3 and C5 for PP and at C2, C3, and C5 for DK met or exceeded these thresholds.

## Discussion

Our analysis of over 5,000 PGY-1 and PGY-2 resident physicians across Japan revealed distinct patterns in how time-at-bedside aligns with specific GM-ITE competency domains. Modest gains were seen for medical interview and professionalism (MP) and for symptomatology and clinical reasoning (CR) domains, whereas enhancements in physical examination and clinical procedures (PP) and disease knowledge (DK) domains were markedly more pronounced. This pattern suggests that domains requiring repeated hands-on practice and knowledge reinforcement benefit most from additional time-at-bedside, whereas complex reasoning and professional behaviors may depend more on qualitative aspects of education, such as targeted feedback and structured reflection.

In our analysis, greater bedside exposure did not correspond to higher scores in the medical interview and professionalism (MP) domain. Although direct patient encounters and observation of attendings’ conduct can model patient-centered practice and interprofessional collaboration [17], these unstructured experiences alone may fall short of instilling professional behaviors. Indeed, critiques of Japanese postgraduate training highlight a gap in formal professionalism curricula [18], which may underlie the weak MP linkage we observed. To truly cultivate resident professionalism, residency programs should integrate time-at-bedside deliberate educational strategies—such as faculty-facilitated teaching rounds, structured feedback checklists, and guided reflective exercises—that explicitly target professional competencies.

We found no significant association between time-at-bedside and symptomatology and clinical reasoning (CR) domain. Instead, residents training at university and affiliated hospitals—where diagnostic imaging and laboratory tests are readily available—tended to score lower in CR [10], suggesting that an overemphasis on advanced diagnostics may detract from logic-driven reasoning. Traditionally, bedside learning has served as the crucible for clinical reasoning by integrating patient interviews, focused physical examinations, hypothesis generation, and management planning [19, 20]. However, the rise of sophisticated diagnostic technologies has gradually transferred much of this process away from the bedside [21]. Modern theories of clinical reasoning highlight the importance of meta-cognitive skills—such as managing uncertainty, reflecting on one’s thought process, and synthesizing complex information—that go beyond procedural steps [22]. Our findings therefore underscore the need to augment bedside exposure with structured educational strategies, including case-based tutorials, targeted feedback from clinical educators, and facilitated reflection sessions. Simulation exercises, problem-solving workshops, and team-based reasoning rounds also show promise for strengthening analytic skills in diverse clinical contexts [19, 20]. Taken together, these results advocate for a balanced, multimodal curriculum that combines hands-on patient care with deliberate pedagogical interventions to foster robust clinical reasoning.

Based on our analysis, more time-at-bedside was associated with meaningful improvements in the physical examination and clinical procedures (PP) and disease knowledge (DK) domains. In the C2, C3, and C5 categories, the observed adjusted score differences in these two domains met or exceeded those differences between PGY-2 and PGY-1—a practical benchmark for clinical significance. This finding suggests that increased bedside engagement may accelerate the development of procedural proficiencies and foundational medical knowledge. In Japan’s PGME, dedicated training in physical examination is often limited [23], which may explain why residents in community hospitals—where time-at-bedside tends to be higher—demonstrate higher PP performance [10]. Moreover, more time-at-bedside may create additional opportunities for direct, hands-on supervision by senior physicians, further reinforcing these competencies [24]. Notably, the PP and DK scores in C4 (90–110 min/day) and C6 (≥ 150 min/day) did not follow the overall stepwise trend. For C4, this deviation may be explained by the relatively small sample size (*n* = 230, 4.3%), which could have reduced the stability of the estimates. In contrast, the results in C6 should be interpreted with particular caution, as the group was very small (*n* = 83, 1.6%) and thus unlikely to provide reliable or generalizable estimates. Therefore, these findings are better considered as outliers without strong educational implications.

Our results offer two key takeaways for postgraduate medical education. First, extended bedside engagement appears to bolster skills in physical examination, clinical procedures, and disease knowledge—effects that were especially pronounced in university hospitals, where baseline procedural proficiency is lower [10]. Although our cross-sectional design precludes definitive causal inferences, the observed adjusted differences align with the notion that hands-on patient contact reinforces these particular competencies. Second, simply more time-at-bedside does not uniformly enhance all domains. Inpatient settings with ready access to imaging and laboratory diagnostics may inadvertently sideline the reasoning process, limiting gains in clinical reasoning (CR) competency [25, 26]. In contrast, rotations in ambulatory clinics—where diagnostic ambiguity persists—offer more fertile ground for honing CR through active hypothesis generation and management planning. This consideration underpinned the 2020 mandate to include outpatient rotations in Japan’s postgraduate medical education curriculum [9].

Additionally, our findings may suggest a potential need for institutional leaders and training program developers to consider strategies to protect and prioritize time-at-bedside. A previous study has highlighted system-level approaches to strengthen bedside teaching and clinical skill acquisition [19]. As health systems worldwide pivot toward patient-centered care models, ensuring sufficient opportunities for bedside learning and clarifying the competencies that benefit from such time may help enhance both clinician proficiency and overall care quality.

Several limitations warrant consideration. First, our reliance on self-reported time-at-bedside may introduce recall bias, as residents might not accurately recollect their daily exposure over two postgraduate years. Although methods such as real-time locating systems or direct time-motion observation improve measurement precision, they are impractical for a nationwide cohort [27, 28]. Notably, 91% of participants reported 10–80 min per day (categories C1–C3)—consistent with prior time-motion data indicating time-at-bedside accounts for about 13% of duty hours—which partially mitigates but does not eliminate this concern [27, 28]. Second, the cross-sectional design precludes causal inferences and raises the possibility of reverse causation residents who demonstrate superior physical examination and clinical procedures (PP) and disease knowledge (DK) competency domains may naturally spend more time-at-bedside. Prospective longitudinal studies are needed to untangle directionality. Third, we lacked details on the clinical context of time-at-bedside—including the specific procedures performed, whether encounters occurred in inpatient versus outpatient settings, and the degree of direct versus team-based interaction. Fourth, while the GM-ITE yields domain-specific scores, it remains a computer-based exam and may not fully capture authentic clinical performance. We adopted the PGY-related score gaps as pragmatic thresholds for clinical significance, but these benchmarks warrant further validation in real-world settings. Previous validation studies have demonstrated acceptable reliability and validity of the GM-ITE overall, particularly in domains such as physical examination, clinical procedures, and disease knowledge. Nevertheless, the assessment of each competency, especially professionalism (MP domain), using computer-based multiple-choice questions has inherent limitations. Professional behaviors are complex and context-dependent, and thus may not be fully captured by this testing format. Fifth, we did not assess downstream effects of time-at-bedside on patient outcomes, patient education, or resident well-being—domains that randomized trials of duty-hour reforms have explored in other contexts [29–31]. Finally, selection bias may limit generalizability, as only 5,344 of 18,188 resident physicians participated in our analysis [32]. Since program directors decide on GM-ITE participation, resident physicians in our analysis may be skewed toward those training at hospitals whose directors are particularly committed to medical education. In addition, one-third of resident physicians were excluded from our analysis, likely reflecting the burden of a 61-item questionnaire.

In summary, we demonstrated that self-reported time-at-bedside is significantly linked to performance in the physical examination, clinical procedures, and disease knowledge competency domains. Future prospective longitudinal studies are needed to clarify whether more time-at-bedside can accelerate the acquisition of these competencies.

## Supplementary Information

Supplementary Material 1.

## Data Availability

Residents who participated in this study did not give consent for their data to be shared publicly. So supporting data is not available. The corresponding author will respond to inquiries on the data analyses in this study.

## Abbreviations

aSD: Adjusted score difference
CI: Confidence interval
CR: Symptomatology and clinical reasoning
DK: Disease knowledge
GM-ITE: General Medicine In-Training Examination
JAMEP: Japan Institute for Advancement of Medical Education Program
MP: Medical interview and professionalism
PGME: Postgraduate medical education
PGY: Postgraduate year
PP: Physical examination and clinical procedure
