# Supplementary material for: Time-at-bedside and competency acquisition: a secondary analysis of GM-ITE domain scores in Japanese resident physicians
Source: BMC Med Educ. 2025 Nov 3;25:1537. doi: 10.1186/s12909-025-08076-9 (PMC12581418; doi:10.1186/s12909-025-08076-9)
Supplement: Supplementary file 1 — Supplementary Material 1. [file 12909_2025_8076_MOESM1_ESM.docx]

**Supplementary Information: Video-Based Question Sample**

**Question:**

A previously healthy 46-year-old man is seeking care for twitching in the left arm. Neurological and other physical findings are normal.

A video showing the findings in the left upper arm is available at the following link:

| 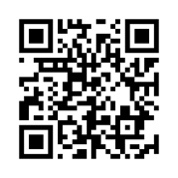 | https://vimeo.com/488752675/6fd2ad2f8a |
| --- | --- |

What disease do these findings suggest?

(A) Amyotrophic lateral sclerosis (ALS)

(B) Antiphospholipid syndrome

(C) Creutzfeldt-Jakob disease

(D) Parkinson’s disease

(E) Lance-Adams syndrome

**Answer:**

(A) Amyotrophic lateral sclerosis (ALS)

**Details:**

When examining a patient with involuntary movements, clinicians must describe the phenomena they observe exactly as the phenomena appear and must also be acquainted with generally known symptoms.

Of the attempted approaches to classification, an approach that classifies movement according to the site involved is relatively easy to grasp. It focuses on (1) movement at the level of the muscle fiber and/or bundle, (2) movement extending to one or several muscles, and (3) movement at the level affecting the limbs and/or trunk. The involuntary contractions seen in the video, which seem to occur in a single muscle and feature no joint movement, are classifiable as “(1) movement at the level of the muscle bundle.” This category includes fasciculation and myokymia.

This type of involuntary movement calls to mind diseases involving lower motor neuron, such as amyotrophic lateral sclerosis (ALS), multifocal motor neuropathy, radiation plexopathy, and Isaacs’ syndrome. In this case, myokymia was electromyographically confirmed. Myokymia is a relatively infrequent electromyographic anomaly in ALS but does appear in clinical practice. Choice 1 is therefore correct.

Antiphospholipid syndrome frequently causes chorea, and Creutzfeldt-Jakob disease is characterized by myoclonus. Although Parkinson’s disease features a variety of involuntary movements, resting tremors are most typical. Lance-Adams syndrome, which follows hypoxic encephalopathy, is action myoclonus caused by cortical excitation.

**Reference**

Whaley NR, Rubin DI. Myokymic discharges in amyotrophic lateral sclerosis (ALS). Muscle Nerve. 2010;41:107–9.
